# E-cigarettes can emit formaldehyde at high levels under conditions that have been reported to be non-averse to users

**DOI:** 10.1038/s41598-018-25907-6

**Published:** 2018-05-15

**Authors:** James C. Salamanca, Jiries Meehan-Atrash, Shawna Vreeke, Jorge O. Escobedo, David H. Peyton, Robert M. Strongin

**Affiliations:** Department of Chemistry, Portland State University, 1719 SW 10th Ave., Portland, OR 97201 USA

## Abstract

E-cigarette aerosol emission studies typically focus on benchmarking toxicant levels versus those of cigarettes. However, such studies do not fully account for the distinct chemical makeup of e-liquids and their unique properties. These approaches often conclude that there are fewer and lower levels of toxins produced by e-cigarettes than by cigarettes. In 2015, we reported the discovery of new hemiacetals derived from the reaction of formaldehyde and the e-liquid solvents. The main finding was that they constituted a significant proportion of potentially undetected formaldehyde. Moreover, unlike gaseous formaldehyde, the hemiacetals reside in the aerosol particulate phase, and thus are capable of delivering formaldehyde more deeply into the lungs. However, the findings were criticized by those claiming that some of the results were obtained under conditions that are averse to vapers. A “reinvestigation” of our study was recently published addressing this latter issue. However, this reinvestigation ignored major details, including no mention of the formaldehyde hemiacetals. Herein, we isolated both gaseous formaldehyde and formaldehyde hemiacetals at an intermediate power level claimed, in the “reinvestigation”, to be relevant to “non-averse,” “normal” usage. The results were that both gaseous formaldehyde and formaldehyde from hemiacetals were produced at levels above OSHA workplace limits.

## Introduction

In 2016, over 9 million Americans were current electronic cigarette (e-cigarette) users^1^, including more than 2 million U.S. middle and high school students^2^. It is thus concerning if even a minority of users cannot properly control e-cigarette-derived intake of formaldehyde and related toxins. Central to this issue is whether an unpleasant taste always accompanies a generic vaper’s exposure to harmful levels of aerosol components. If true, then e-cigarette users could self-regulate toxicant intake by simple adjustments, such as lowering the device power level, until a pleasant flavor is achieved. Vapers are encouraged to use power levels at just below those that are unpleasant (e.g., burnt) in taste, as justified in specific recent reports claiming that toxic levels of aldehydes are found solely in unpleasant-tasting aerosols^3–5^.

**Figure 1:**
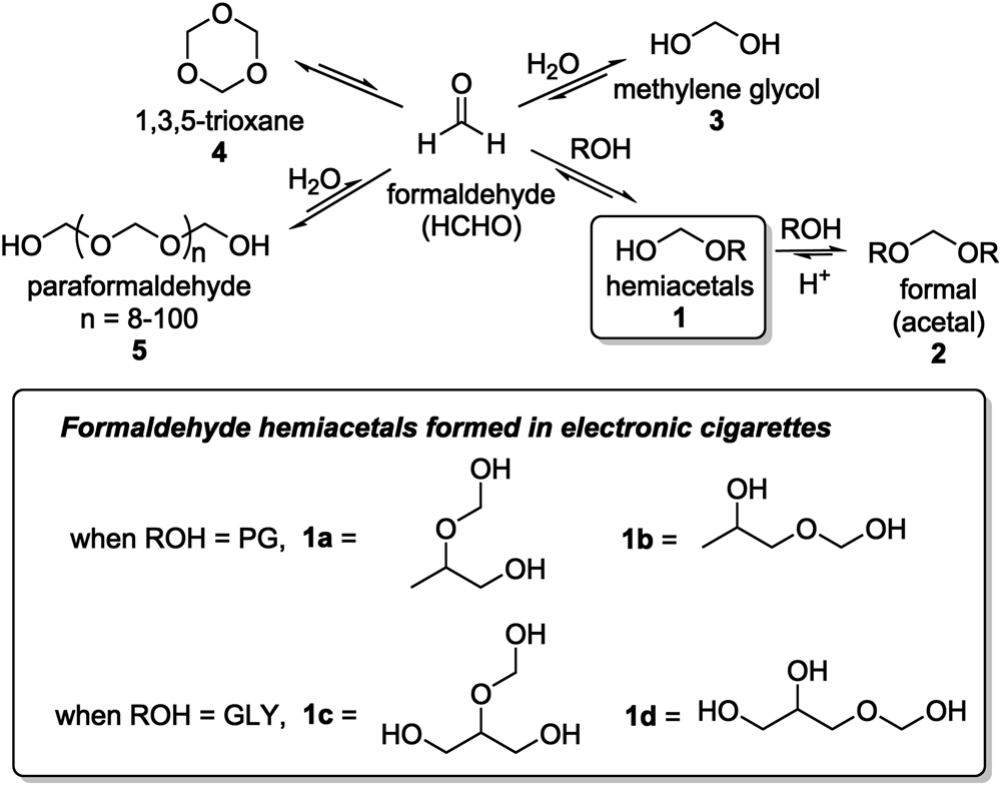
Gaseous formaldehyde (HCHO) and examples of its common equilibria. Balashov^7^ found that **1** is the major species formed in the equilibrium between HCHO and common alcohols. He found that formaldehyde hemiacetals were the major species in solutions of formaldehyde containing excess alcohol. When water the main solvent, the equilibrium involves mainly formaldehyde and methylene glycol/paraformaldehyde. Compounds **1a-d** were reported as major e-cigarette aerosol components by us in 2015^6^.

In 2015, we reported the discovery of new hemiacetal derivatives of formaldehyde (**1a-d**, Fig. 1) in electronic cigarette aerosols, formed via the reaction of gaseous (carbonyl) formaldehyde (HCHO) with propylene glycol (PG) and glycerol (GLY)^6^. The HCHO in e-cigarettes is initially formed via the degradation of PG and GLY. Importantly, **1a-d** were the major components present in the ^1^H NMR spectrum of the crude (unprocessed) aerosol material, present at levels of up to *ca*. 2% of the amount of PG and GLY solvent in the aerosol samples collected.

The formation of significant amounts of **1a-d** is consistent with the chemical literature. For example, formaldehyde hemiacetals were shown to be the most abundant components (e.g., *ca*. 90% or more) found in investigations of equilibrium mixtures of formaldehyde and various alcohols (Fig. 1), including the prototypical 1,2-diol, ethylene glycol^7^. We examined **1a-d** formation at both high power (5 V) and low power (3.8 V) e-cigarette settings. The low power setting led to no detectable hemiacetal formation via ^1^H NMR. To put the relatively large amount of **1a-d** formed at higher power settings in context, a cancer slope factor was calculated and compared to that of free formaldehyde levels present in traditional cigarette smoke. This was of course based on the stated assumption that all of **1a-d** converts to carbonyl HCHO. In contrast, we also reported that intact **1a-d** would be of concern, since they would deposit more efficiently in the respiratory tract compared to gaseous HCHO^6,8^. Even at the highest total particulate matter levels for e-cigarette aerosols, formaldehyde as gaseous HCHO will be delivered to the user in the gas phase. The formaldehyde hemiacetals, however, will be delivered mostly via the deposition of aerosol droplets. The partitioning of a molecule into the gas or PM phase will determine how it is deposited in the respiratory tract^9–11^.

Compounds **1a-d** have since been shown to form at levels higher than those of gaseous HCHO in e-cigarette aerosols. Moreover, **1a-d** have not been fully accounted for in studies claiming that vapers can self-regulate the intake of formaldehyde by sense perception. An example is a recent paper by Farsalinos and co-workers, termed a “replication study” of our 2015 investigation, using the same brand of atomizer (CE4 top coil atomizer) and e-cigarette liquid (e-liquid, Halo Café Mocha)^4^. Despite the fact that our manuscript reported the determination of only **1a-d**, and did not include gaseous HCHO, the “replication study” never mentions hemiacetals **1a-d**. In addition, they used a more aggressive puffing regimen (50 puffs/sample) as well as DNPH impingers for derivatization and HPLC analysis^4^. In contrast, in 2015, we used non-destructive ^1^H NMR (10 puffs/sample) without any aerosol sample processing, except for dilution in an NMR solvent^6^. Importantly, we have shown that levels of **1a-d** are not accurately determined using DNPH impingers or DNPH sorbent cartridges^12^. Their main purpose was to prove that formaldehyde would only form at levels of concern to human health under unpleasant sensorial conditions due to overheated liquid and/or a dry heating coil (colloquially known as a “dry puff”), and therefore would be readily detectable by users^3–5^. The distribution of reported voltage settings by which dry puffs are detected are subjective across various users including those with extensive vaping experience. The study does not address the relationship between the level of e-cigarette usage experience and the effects of unpleasant aerosol tolerance as it pertains to e-cigarette exposure^13^. Flavorants and nicotine may also have varying degrees in masking or accentuating unpleasant aerosol experiences that can affect the taste detectability of dry puff conditions. These layers of unaddressed complexities undermine the premise of studies reinvestigating hemiacetals utilizing human reported safe levels of use. Herein, we show that not only **1a-d** but also gaseous HCHO form at concerning levels at an intermediate CE4 device power setting, the same setting defined recently^4^ as appropriate to real-world human usage.

## Methods

### Aerosol generation

A Single Cigarette Smoking Machine (SCSM-STEP, CH Technologies), was calibrated to a puffing regimen of 60 mL puff volume, 4 s puff duration and a 30 s inter-puff interval. Aerosol was generated using a CE4 top coil atomizer (Central Vapors) with an Innokin iTaste VV V3.0 variable voltage battery. Two new CE4 atomizers were used at 4.0 V. The atomizer was weighed before and after vaping to determine the e-liquid consumed per experiment (Table S1, Supporting Information). Aerosol generated from 50 puffs was collected, after which 3 blank puffs were drawn through the machine to assure the tubing was cleared of all aerosol prior to dismantling. The e-liquid used was Halo Café Mocha (Nicopure Labs LLC) with a nicotine concentration of 6 mg/mL. The CE4 device, and the e-liquid (Café Mocha) were chosen solely to enable comparison to the relevant prior reports^4,6^.

### Sample collection

The aerosol collection setup (Fig. S1, supporting information) consisted of two cold traps chilled to −77 °C using isopropyl alcohol and dry ice which were connected in series to two impingers filled with 20 mL of a 12.2 mM solution of dinitrophenylhydrazine (DNPH, purified by recrystallization) and 146 mM of phosphoric acid in 1:1 HPLC-grade water and HPLC-grade acetonitrile (Honeywell), as per the CORESTA standard method for carbonyl trapping. The first cold trap was connected to the e-cigarette by 5 cm ACF0027-F Tygon S3 E-3603 tubing. The pair of cold traps were connected by 2.5 cm tubing, and the second cold trap and the first DNPH-impinger were connected by 5 cm tubing. The pair of impingers were connected by 3 cm tubing. The second impinger and the SCSM were connected by 1.5 cm tubing. Laboratory air was sampled and analyzed for potentially interfering levels of analytes.

### Sampling and NMR analysis

After the conclusion of each experiment, the cold traps were allowed to warm to room temperature. They were extracted with 10 mL of HPLC-grade acetonitrile which was spiked with 900 μL of a 2.03 mM solution of 1,2,3,4-tetrachlorobenzene (Sigma-Aldrich), used as an internal standard to account for losses in the extraction process (60–70% yield). The rinsate was collected and concentrated via rotary evaporation at 25 °C and 60 Torr. The residue was dissolved in a 2 mL volumetric flask using DMSO-*d*_6_ + 0.05% tetramethylsilane (99.9% D, Cambridge Isotope Labs) NMR solvent containing 26 μL of HPLC-grade H_2_O (Honeywell) and an NMR quantification standard, 2,3,4,5-tetrachloronitrobenzene (TCI Chemicals, 902 μM). The H_2_O was added to the NMR sample to enable better resolution of the –OH proton peaks of **1a-d** from an overlapping resonance.

NMR spectra were collected using 512 scans, a 6.7 second repetition rate, and a 30-degree flip angle on a Bruker Avance III 600 MHz NMR spectrometer. Spectra were processed using 0.3 Hz line broadening with a final data size of 64 k real data points. The levels of **1a-d** were determined using Global Spectral Deconvolution from MestreLab software.

### Sampling and HPLC analysis

The contents of each impinger were dissolved in HPLC-grade acetonitrile and combined in a volumetric flask. This solution was diluted with acetonitrile to 50 mL total volume. The HPLC syringe and injection port were rinsed 3x with HPLC-grade methanol and acetonitrile, and injected with 20 μL of sample. The HPLC system consisted of a 1525 Binary HPLC Pump with a 2996 Photodiode Array Detector (Waters Inc.). The stationary phase consisted of a pair of SUPELCOSIL C-18, 25 cm × 4.6 mm, 5 μm particle size columns (Supelco) connected in series and heated at 40 °C. The mobile phase comprised of acetonitrile/water with a gradient system as follows: 0 min. 60/40; 7 min. 60/40; 25 min. 100/0, at a combined flow rate of 1 mL/min, with 360 nm detection wavelength. The sample injection volume was 20 μL.

## Results and Discussion

### Choice of power setting

The “upper limit of realistic usage,” of the CE4 product was recently suggested as 4.0 V (7.3 W), based on the subjective perceptions of vapers^4^, in a human subjects study included in the “reinvestigation” of our 2015 report^6^. However, the study was not completely blinded. Subjects were asked if they had knowledge of and ability to detect “dry puffs,” the experimental endpoint. Up to seven distinct power levels were increased during the vaping sessions in order, from lowest to highest power, rather than in a randomized manner. The justification was that experienced vapers in the study could detect the higher power levels due to their perception of concomitantly larger inhaled aerosols, so it was suggested that there was no need to randomize. However, this assumption contradicts the researchers’ own data^4^, as well as earlier findings by others^14^ showing that aerosol volumes can contract at higher power levels when using the CE4 e-cigarette. Despite the concerns, herein we note the findings of the “replication study”, defining 4.0 V (7.3 W) as the recommended “safe” power setting.

### Collecting and analyzing HCHO and 1a-d from the same sample

Recently, we described a tandem cold-trap/DNPH impinger/smoking machine set-up for separating and trapping both aerosol particulate matter as well as gas-phase aldehydes derived from the same puff^12^. The aerosol initially is pulled through a pair of cold-traps (−77 °C) that aid in inhibiting possible unwanted further reactions, allowing for effectively trapping relatively unstable materials such as intact **1a-d**. In the current study, we added additional washes of the cold-traps with acetonitrile to improve the recovery accuracy of **1a-d**. The NMR quantification standard versus the internal standard signal ratio was used to calculate the yield of the acetonitrile extraction. The amount of formaldehyde hemiacetal observed in the spectra was subsequently factored up to account for transfer losses. No gaseous HCHO is found in the cold-traps^12^, which is instead found as its DNPH adduct in the impingers, connected in series after the cold traps. The impingers were set up according to the CORESTA recommended method, as described previously^12^. The aerosol components found in the cold-traps were dissolved and analyzed by ^1^H NMR. The HCHO-DNPH adducts formed in the impinger were analyzed by HPLC.

### Aerosol levels of 1a-d are formed in several-fold excess of those of gaseous HCHO

Table 1 shows that the levels of **1a-d** are formed in excess compared to those of gaseous HCHO. This is in agreement with our investigations using other e-cigarettes^12,15^. Interestingly, the amount of **1a-d** found in the current investigation is higher, in each of the four runs, compared to our initial 2015 study (380 μg/10 puffs), despite the higher power (5 V) used in the older study^6^. One reason the levels of **1a-d** are higher herein is that we used the harsher 50 puff “replication study” puffing regimen, which the researchers performing the latter study had modified^4^ (without explanation) from our original milder 10 puff experiments. In addition, in our prior investigation^6^, the aerosol was pulled from the e-cigarette manually via syringe, collected as it passed over the surface of DMSO-*d*_6_ in an NMR tube, at room temperature. One could visibly see a significant amount of aerosol lost to the ambient atmosphere using this older method, prompting us to state in the 2015 paper that the levels of **1a-d** were underestimated^6^. Others have independently replicated the use of this latter syringe and non-destructive ^1^H NMR method in a relatively newer e-cigarette model, and found that **1a-d** comprised a significant percentage of the total formaldehyde in the aerosol of a different e-cigarette^16^.

**Table 1:** Levels of **1a-d**, HCHO and e-liquid consumed (commercial Café Mocha brand, the same used in our 2015 study, ref.^6^) from four vaping sessions with two CE4 atomizers at a power level of 4.0 V.

	Run	**1a-d** (µg/10 puffs, as HCHO equivalents)	Gaseous HCHO (µg/10 puffs)	HCHO + **1a-d**, (µg/10 puffs)	E-liquid consumed/puff (mg)
CE4 atomizer 1	1	669	159	828	6.0
	2	1147	138	1285	6.0
CE4 atomizer 2	3	816	103	919	6.6
	4	677	138	815	5.4
	Average	827	135	962	6
	95% CI	356	37	351	1

Several-fold more **1a-d** is produced compared to HCHO. The *p*-value is 0.00049. The result is significant at *p* < 0.05. The levels of **1a-d** are reported for 10 puffs (for comparison to literature values), but were obtained from 50 puff vaping sessions.

### The levels of gaseous HCHO and 1a-d in the context of the recent literature

In addition to Farsalinos *et al*.^4^, Gillman and co-workers have also investigated the formation of formaldehyde in a CE4 device (Table 2)^14^. They found that the CE4 emitted formaldehyde levels that were above OSHA (TWA) workplace limits, as well as above those from 20 traditional cigarettes. Importantly, these concerning levels of formaldehyde were observed at every CE4 power level used, including those below the 7.3 W (4.0 V) “defined” safe threshold^4^, and even at the lowest power level studied (5.3 watts)^14^.

**Table 2:** Comparison of formaldehyde levels in the current and two recent studies using a CE4 e-cigarette.

Power (Watts)	Formaldehdye levels (μg/puff)		
	Farsalinos *et al*.^4^	Gillman *et al*.^14^	Study herein^c^
5.0	0.34 ± 0.22	ND	ND
5.3	ND	8.5 ± 8.9^b^	ND
6.5	ND	21 ± 16^b^	ND
7.3	1.98 ± 0.56	ND	13.5 ± 3.2
7.8	ND	32 ± 12^b^	ND
8.0	10^a^	ND	ND
9.2	ND	51 ± 31^b^	ND
11.4	71.82 ± 5.82	ND	ND

The two cited studies^4,14^ did not involve separating and quantifying **1a-d** and gaseous HCHO separately, and may therefore represent a mixture in which **1a-d** partially converted to HCHO prior to DNPH-trapping. The results shown from the study herein correspond to only gaseous HCHO levels for comparison. ND = not described.

^a^Reported in ref.^4^ as 100 μg/10 puffs. ^b^E-liquid was composed of 48% PG in GLY and 2% nicotine, and formaldehyde values were obtained using a CE4 atomizer with an Innokin iTaste VV4 battery. ^c^HCHO levels correspond to those in Table 1.

Intriguingly, there is a >10-fold discrepancy between the levels of formaldehyde reported between the Farsalinos^4^ and Gillman groups^14^, within the 6.5–7.3 W range (Table 2). However, it should be noted that Gillman used a different e-liquid. Moreover, neither of these two studies explicitly accounted for levels of **1a-d**. Our finding of 13.5 ± 3.2 μg of gaseous HCHO/puff at 7.3 W embodies an intermediate value of formaldehyde. It is above the OSHA (TWA) workplace limits (5.3 mg/day)^14^, calculated as described previously (13.5 μg HCHO/puff ÷ 6 mg aerosol/puff = 2.25 mg gaseous HCHO/g aerosol)^14^. Using a conservative value of 4 g e-liquid consumed/day^17^ affords 10.0 mg gaseous HCHO inhaled/day, nearly double the OSHA limit.

### The levels of gaseous HCHO and 1a-d in the context of self-regulation of toxin intake

In the “replication study,” which involved human subjects (Table 2, first column), it was reported that, at the 4.2 V (8 W) power level, 88% of participants detected “dry puffs”^4^. This means that 12% of the subjects would have been exposed to *ca*. 10 μg of formaldehyde/puff at 4.2 V (8 W)^4^, without any sensory awareness that they should cease usage at an exposure level that corresponds to a daily intake of 8.3 mg of formaldehyde per day, which is above the OSHA limit. Notably, this was calculated using the data from the relatively lowest reported levels (those from ref.^4^, the “replication study”). The levels of gaseous HCHO obtained in the current study are approximately six-fold higher at lower power (7.3 W, 4.0 V), without factoring in any contribution to total formaldehyde from **1a-d**. The levels of formaldehyde from Gillman’s study^14^ were 2- and 3-fold higher than those of ref.^4^ even when obtained at the relatively lower power levels of 6.5 W and 7.8 W (Table 2).

The inconsistencies in the inter-laboratory data displayed in Table 2 is in keeping with the literature, and not just in studies that have involved CE4 e-cigarettes. Several researchers, including us, have noted elsewhere the concerning interlaboratory differences in reported e-cigarette toxin levels as well as the factors exacerbating this issue^18–22^. When care is taken to avoid the drying of heating coils and/or burning e-liquids in laboratory studies, such as using single puff samples^15^, using power levels described by vapers as popular for specific devices^19^ and/or applying settings corresponding to manufacturers’ recommendations^23^, etc., wide variations in levels of aerosol toxins can still be observed. Interestingly, in a recent, related “re-investigation” of work by Sleiman *et al*., Farsalinos and Gillman reported that, even under “dry puff” conditions, they found formaldehyde levels that were not only below OSHA thresholds but also were >12-fold lower than those reported in the original manuscript by Sleiman (under the same conditions)^5^. Thus, measurements of toxicant levels can differ by orders of magnitude between labs for reasons other than “dry puff”; moreover, “dry puff” conditions should not always be cited as the sole factor promoting elevated levels of carbonyls.

### Limitations

Limitations of the study herein include the fact that we did not use human subjects. However, using e-cigarette data acquired based on recommended settings, as opposed to concomitant human testing, is well-precedented, for example, by the same authors^23^ who published the “replication study”^4^ of our 2015 findings. Regardless, had we reported the results described herein at the intermediate 4.0 V power level three years ago, it would have been concluded by those performing the “replication study”^4^, that they were obtained under “normal,” “non-averse” vaping conditions. Importantly, even if one assumes the unlikely scenario wherein the CE4 device produced all “dry-puffs” at 7.3 W in every experiment (Table 1) in the current study, thereby inflating the levels of HCHO and **1a-d** under conditions “averse” to users, the results of the “replication study”^4^ show that a concerning percentage (12%) of human subjects could not detect dry puffs under conditions affording levels of formaldehyde that are above the OSHA threshold. Moreover, the aforementioned results do not account for the facts that the harsh taste of formaldehyde and other aldehydes is dulled due to the nicotine drive^24^, as well as by the cross-desensitization of transient receptor potential ankyrin subtype 1 (TRPA1) channels in sensory neurons^25,26^.

## Conclusion

We have revisited our 2015 investigation^6^ using an improved sample collection method^12^ that separates particulate- and gas-phase e-cigarette aerosol formaldehyde components from the same puff. In addition, an intermediate power setting was chosen compared to our prior work, one that was purported to represent “normal” vaping conditions^4^. Both HCHO and **1a-d** levels were found in the aerosol at levels above the OSHA guidelines. Our work continues to show that formaldehyde hemiacetals, a new form of formaldehyde, may serve as delivery agents that deposit more deeply in the lungs compared to gaseous formaldehyde. This main finding, the discovery of **1a-d**, from our prior studies, was ignored in the recent “replication study”^4^. The variability in the subjective evaluation of aerosols by users^4^, as well as factors influencing discrepant interlaboratory levels of emissions, are significant current issues in the e-cigarette field that require extensive further study.

## Electronic supplementary material

Supporting Information
