# Supplementary material for: E-cigarettes can emit formaldehyde at high levels under conditions that have been reported to be non-averse to users
Source: Sci Rep. 2018 May 15;8:7559. doi: 10.1038/s41598-018-25907-6 (PMC5954153; doi:10.1038/s41598-018-25907-6)
Supplement: Supplementary file 1 — Supporting Information [file 41598_2018_25907_MOESM1_ESM.pdf]

E-cigarettes can emit formaldehyde at high levels under conditions that have been reported to be non-averse to users

James C. Salamanca, Jiries Meehan-Atrash, Shawna Vreeke, Jorge O. Escobedo, David H. Peyton, and Robert M. Strongin*

Department of Chemistry, Portland State University, 1719 SW 10th Ave., Portland, OR, 97201, USA

Correspondence to strongin@pdx.edu

Supporting Information

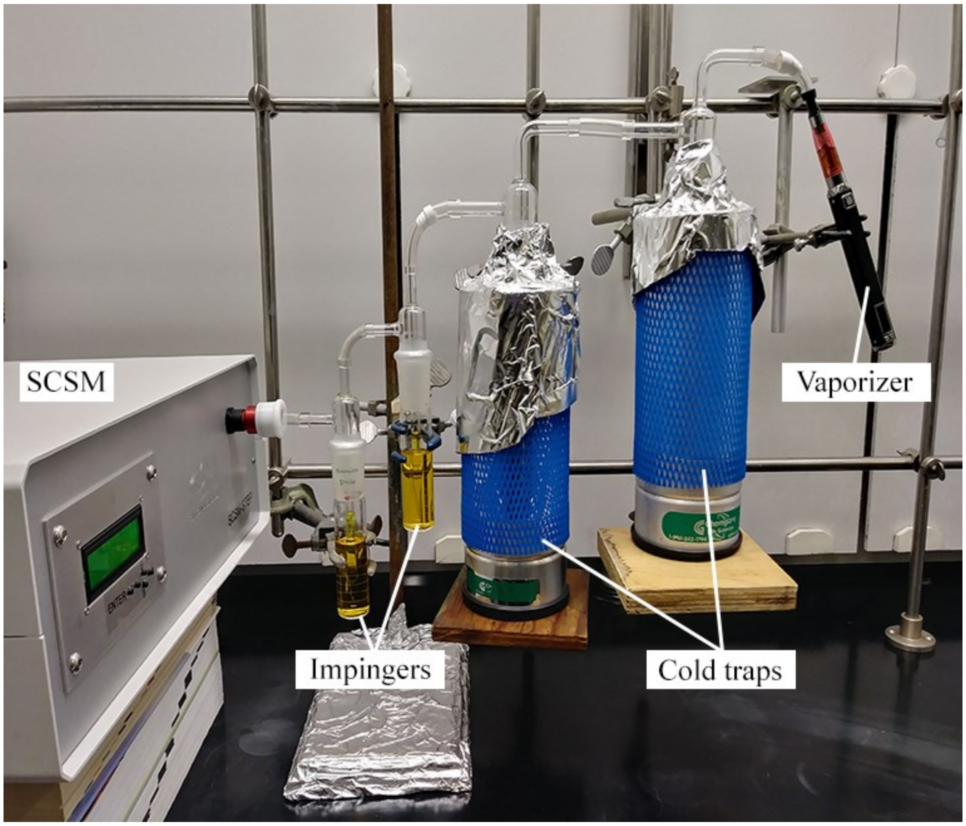

Figure S1: Vapor collection setup used for experiments herein.

Table S1. Mass of e-liquid consumed during aerosolization.

Experiment Number	Atomizer Tare (g)	Atomizer Post Vape (g)	E-liquid consumed (mg)
1	11.97233	11.67262	299.71
2	12.04055	11.74348	297.07
3	11.43662	11.10536	331.26
4	11.58817	11.31875	269.42

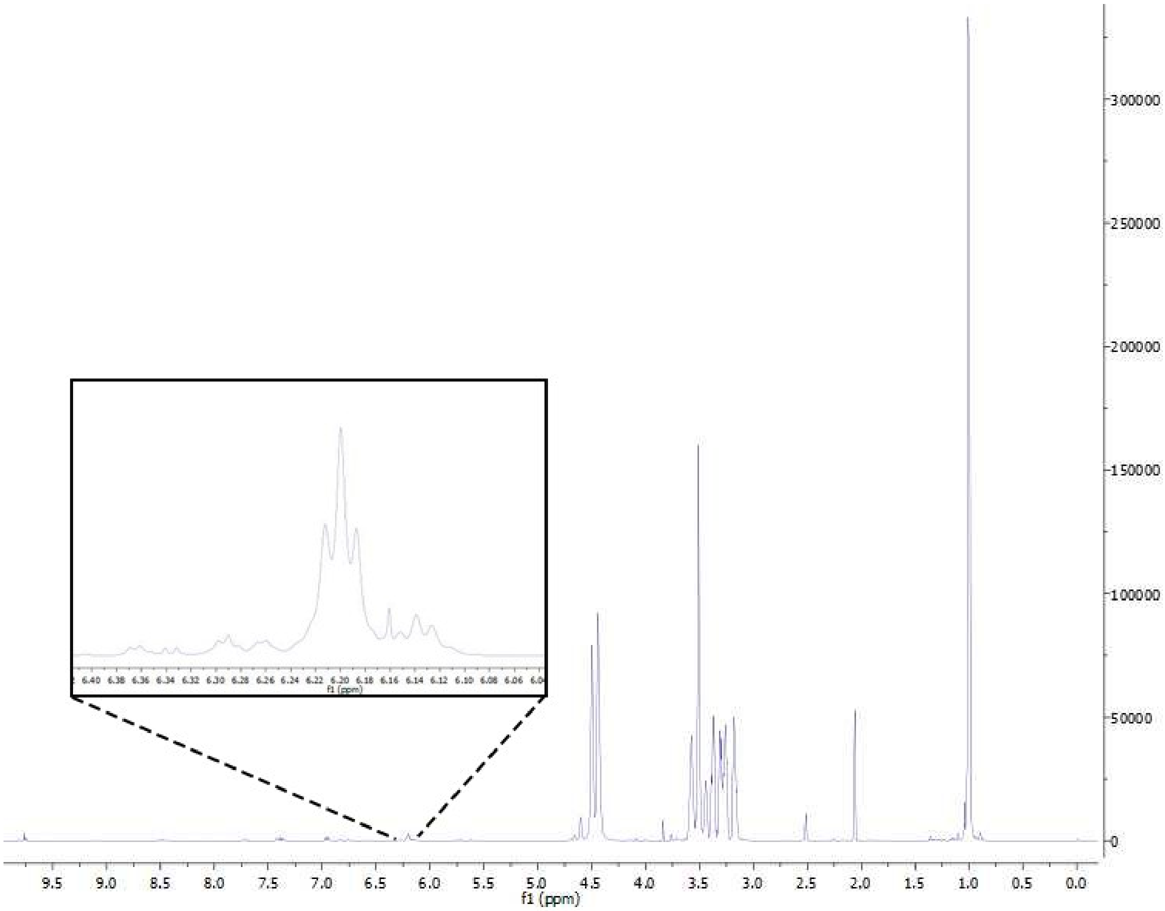

Figure S2: Representative NMR spectrum of a sample collected with the setup shown in Figure S1. An expansion of the region from 6.0 - 6.4 ppm shows the hydroxyl proton resonances related to the hemiacetal (-O-CH_2-O**H**).
